# Adult Sibling Tension and Older Mothers’ Psychological Well-Being: The Moderating Role of Caregiving

**DOI:** 10.1093/geroni/igab046.351

**Published:** 2021-12-17

**Authors:** Megan Gilligan, J Jill Suitor, Yifei Hou, Barbra Brottman, Jeenkyoung Lee, Eunbea Kim

**Affiliations:** 1 Iowa State University, Iowa State University, Iowa, United States; 2 Purdue University, Purdue University, Indiana, United States; 3 Purdue University, West Lafayette, Indiana, United States; 4 Iowa State University, Ames, Iowa, United States

## Abstract

The life course perspective concept of “linked lives” suggests that the lives of adult children and older parents are interconnected and consequential for the well-being of members of both generations. In this work, we consider the association between tension among adult siblings and older mothers’ psychological well-being. We focus specifically on tension in the adult sibling relationship because research has shown that negative relationship quality is especially consequential for well-being. We consider this association in the context of caregiving because this is a time when offspring are often required to coordinate with each other to provide assistance. We utilized data from 304 older mothers (average age = 78) and 736 of their adult children (average age = 49) from the Within-Family Difference Study (WFDS) II. First, we examined the direct association between adult sibling tension and mothers’ reports of depressive symptoms. Second, we examined whether the association between sibling tension and mothers’ depressive symptoms was moderated by mothers’ need for care. Preliminary results indicated no direct effect of sibling tension on mothers’ depressive symptoms. However, moderation analysis revealed that sibling tension was associated with an increase in mothers’ depressive symptoms among mothers who reported needing assistance. These findings highlight the importance of understanding the interconnected nature of adult family relationships especially in the context of later-life family caregiving. In particular, the findings reveal that older mothers in need of care are especially vulnerable to tension in the relationships among their adult children.

